# Production and purification of polyclonal antibody against F(ab')2 fragment of human immunoglobulin G

**Published:** 2017-12-15

**Authors:** Hadi Nasiri, Zahra Valedkarimi, Leili Aghebati-Maleki, Jalal Abdolalizadeh, Tohid Kazemi, Mojghan Esparvarinha, Jafar Majidi

**Affiliations:** 1 *Immunology Research Center, Tabriz University of Medical Sciences, Tabriz, Iran; *; 2 * Department of Immunology, Faculty of Medicine, Tabriz University of Medical Sciences, Tabriz, Iran; *; 3 *Student Research Committee, Tabriz University of Medical Sciences, Tabriz, Iran; *; 4 *Drug Applied Research Center, Tabriz University of Medical Sciences, Tabriz, Iran.*

**Keywords:** F(ab')_2_ fragment, Immunoglobulin G, Pepsin digestion, Polyclonal antibody, Purification

## Abstract

Antibodies are essential tools of biomedical and biochemical researches. Polyclonal antibodies are produced against different epitopes of antigens. Purified F(ab')_2_ can be used for animal’s immunization to produce polyclonal antibodies. Human immunoglobulin G (IgG) was purified by ion exchange chromatography method. In all stages verification method of the purified antibodies was sodium dodecyl sulfate-polyacrylamide gel electrophoresis (SDS-PAGE). Purified IgG was digested by pepsin enzyme and F(ab')_2_ fragment was purified by gel filtration separation method. For production of polyclonal antibody, rabbit was immunized by purified F(ab')_2_ and antibody production was investigated by enzyme-linked immunosorbent assay. Purified anti-IgG F(ab')_2_ was conjugated with fluorescein isothiocyanate. Ion exchange chromatography purification yielded 38 mg of human IgG antibody. The results of SDS-PAGE in reduced and non-reduced conditions showed bands with 25-30 kDa molecular weight (MW) and 50-kDa respectively and a distinct band with 150 kDa MW. The results of non-reduced SDS-PAGE for determining the purity of F(ab')_2_ fragment showed one band in 90 kDa and a band in 150 kDa MW position. Purification by Ion exchange chromatography method resulted about 12 mg rabbit polyclonal antibody. Flow cytometry showed generated polyclonal antibody had an acceptable activity compared to commercial antibody. Taking together, purified IgG F(ab')_2_ and polyclonal anti-IgG F(ab')_2_ are useful tools in biomedical and biochemical researches and diagnostic kits.

## Introduction

Antibodies (Abs) have emerged as essential tools of biomedical researches and are of great commercial and medical values. They are the fastest growing product segments of the pharmaceutical industry. Therapeutic Abs are important drugs for the treatment of cancers, autoimmune diseases and infections. Aside from therapeutic applications, Abs are important tools in diagnosis and medical researches.^1^^-^^3^ Polyclonal antibodies (PAs) are mixtures of monoclonal Abs that are produced against different epitopes of antigens and have great avidity to polyvalent antigens.^4^^,^^5^ Owing to greater avidity to a polyvalent antigen, they have several applications in the case of bacterial agglutination and hemagglutination, complement mediated lysis and for the preparation of immunoaffinity columns as ligands or coupling reagents for binding and detection of molecules in a sample in a variety of assays.^6^

Enzyme-cleaved Abs are widely used for animal immunization and treatment of envenoming. Such products should comprise only highly pure immuno-globulin fragments since Fc or other contaminating fragments may lead to side effects. The production of Abs fragments often involves several steps designed to reduce the side effects while retaining their effectiveness.^7^^,^^8^

The Abs fragments can be used in research and diagnosis. Accordingly, immunoglobulin G (IgG) F(ab')_2_ fragment can be used in diagnostic kits of human diseases such as rheumatoid arthritis and tumors. The F(ab')_2_ fragment has many therapeutic applications such as passive immunotherapy for influenza and radioimmuno-therapeutic agent for leukemia.^8^^-^^11^

The aims of this study include production, purification and fluorescein isothiocyanate (FITC) conjugation of rabbit anti-human IgG F(ab')_2_ fragment. To approach these goals, generation and characterization of a highly specific PA against human IgG F(ab')_2_ fragment were investigated. Produced PA against human IgG F(ab')_2 _can be used for development of biomedical research and diagnostic kits and standardization of this product towards self-sufficiency of the country.

## Materials and Methods

**Purification of IgG. **The IgG used in this study was purified from the pooled human sera by ion exchange chromatography method on a diethylaminoethyl (DEAE)-sepharose column (Pharmacia, Uppsala, Sweden). The column was equilibrated by 40 mM Tris-phosphate buffer (Merck, Darmstadt, Germany) at pH 8.10. Elution of IgG was performed by 75 mM Tris-HCl buffer (Merck) at pH 8.10. Finally, the column was washed by 1 M NaCl (Merck). The flow-rate was 0.50 mL min^-1^. The collected fractions were analyzed by sodium dodecyl sulfate-polyacrylamide gel electrophoresis (SDS-PAGE) under non-reducing and reducing conditions at neutral pH according to the manufacturer's instructions. The concentration of polyacrylamide solution was 12.00%. Samples were boiled with 2.00% SDS (Merck) for 10 min and 0.20 μg of them were loaded for each sample well onto an electrophoresis gel in a vertical chamber. Electrophoresis was done in a mini-PROTEAN^®^ electrophoresis instrument (Bio-Rad Laboratories, Hercules, USA). After separation, the antibodies were stained with Coomassie Brilliant Blue G 250 (Sigma, Deisenhofen, Germany) and imaged.^12^

**Enzyme digestion. **Human IgG was used for digestion with enzyme to give a ratio of 60 mg per 1 mg of pepsin. In this study the optimum pH for pepsin digestion was chosen (pH: 3.20). Then, 3000-unit pepsin enzyme (Sigma) was added, mixed and incubated for 60 min at 37 ^°^C. The enzyme activity was stopped with the appropriate pH inactivation method. Digestion stopped by adding 200 μL of 0.50 M Na_2_HPO_4_ to raise the pH to ~ 8.00.^7^

**Purification of F(ab')**_2_ **. **Gel filtration separation of digested material was performed using G100 matrix (Pharmacia) and normal saline buffer containing 75 mM ethylenediaminetetraacetic acid (EDTA, Merck). The used flow rate was 2 mL per 3 min. In all stages, protein concentration was determined by ultraviolet (UV) spectrophotometer at 280 nm (model 2138 Uvicords; Pharmacia).^13^ Purity of the eluted fractions from the gel filtration column was checked by SDS-PAGE analysis that was performed under reducing conditions according to the standard method. The total polyacrylamide concentration was 12.00% for separating gel and 4.00% for stacking gel. 

**Rabbit immunization and serum preparation. **New Zealand white rabbit was chosen for this study. All procedures were performed according to the guidelines approved by the Ethics Committee of Tabriz University of Medical Sciences, Tabriz, Iran (Appendix 4-9, 1395). The first inoculation was done by 300 μg 300 μL^-1^ of F(ab')_2 _and equal volume of freund's complete adjuant (Sigma) via subcutaneous and intramuscular routes. The second and third inoculations were done intramuscularly using freund's incomplete adjuant (Sigma) on days 21 and 35. Final immunization was done without any adjuant on day 45. Then, Ab production was investigated by designing enzyme-linked immunosorbent assay (ELISA).^14^ The total protein concentration of sera was determined by UV spectro-photometer at 280 nm (Pharmacia). Rabbit sera were centrifuged, precipitated and finally dissolved in 1.50 mL phosphate-buffered saline (PBS; Sigma) and dialyzed against PBS pH 7.20.^15^^-^^18^

**Purification of PA. **The precipitated and dialyzed rabbit anti-human F(ab')_2 _Ab applied to an ion-exchange column on a DEAE-sepharose (Pharmacia). The flow rate was 0.50 mL min^-1^. Equilibration buffer was Tris-phosphate buffer (pH: 8.10; Merck). The elution stage was done by 75 mM Tris-HCl buffer at pH 8.10. The collected fractions were analyzed by SDS-PAGE.^4^^,^^19^

**Conjugation of PA with FITC. **The Ab precipitate was dissolved in PBS (pH: 7.40) and dialyzed against reaction buffer (500 Mm Carbonate, pH=9.20) in 24 hr. One mg FITC (Sigma) was dissolved in 1 mL anhydrous dimethyl sulfoxide (Sigma) before use. The FITC was added to give a ratio of 60 μg per 0.50 mg of IgG and mixed immediately. The product was dialyzed at 4 ^°^C (overnight). Unreacted FITC was removed and the Ab exchanged into Storage Buffer (150 mM NaCl, 10 mM Tris, 0.10% NaH3, pH=8.20).^1^^,^^20^

**Determination of conjugated IgG reactivity. **Reactivity of Ab was assessed using flow cytometry. The data were processed using Cell Quest 3.10 software (Becton-Dickinson, San José, USA).

## Results

**Fig. 1:**
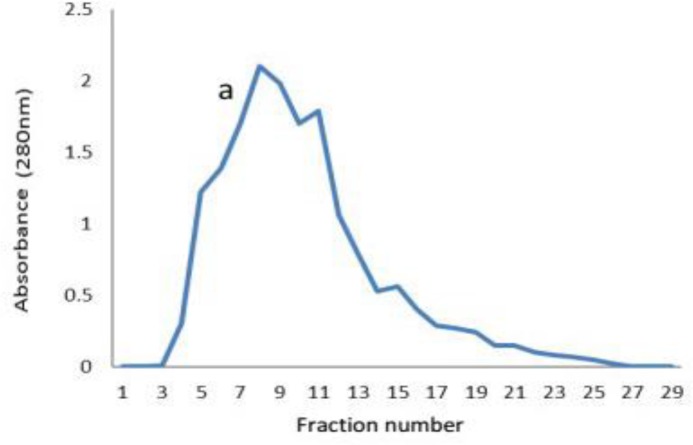
Isolation of human IgG by ion-exchange chromatography. a: separation of IgG by 75 mM Tris-HCl buffer, pH 8.10.

**Fig. 2:**
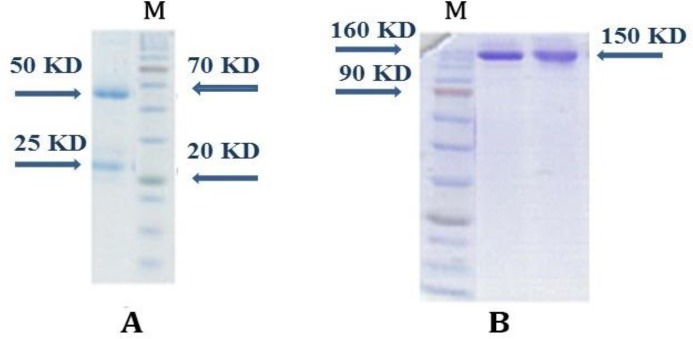
SDS- PAGE results for purity determination of purified human IgG. A) Reduced condition; B) Non-reduced condition.

**Human IgG purification and purity determination. **Protein contents of the human sera (5 mL) were 100 mg. Protein content reduced to 90 mg after preparation stages such as ammonium sulfate precipitation and dialysis. Ion exchange chromatography purification yielded 38 mg of IgG Ab. Purification pattern is shown in Figure 1. The results of reduced SDS-PAGE for determining the purity of purified IgG showed a distinct band in 50 kDa MW position corresponds to Ab heavy chain and the band in 25 kDa MW position corresponds to IgG light chain (Fig 2A). In non-reduced SDS-PAGE results, the 150 kD MW band was demonstrated the purification of whole Ab (Fig 2B).

**Fig. 3:**
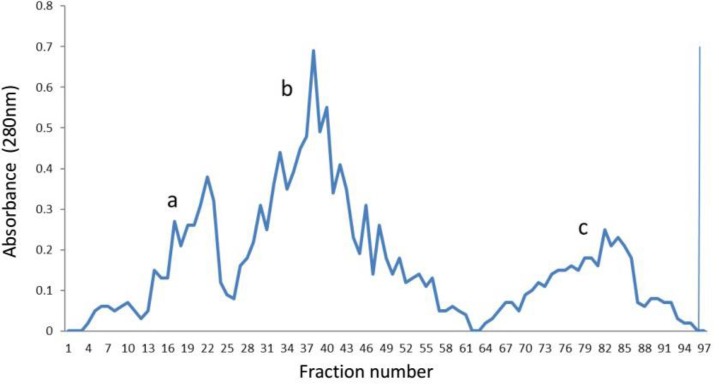
The step wise elution from the gel filtration column. a: separation of IgG; b: elution of F(ab)_2 _fragment by normal saline buffer containing 75 mM EDTA; c: elution of P(Fc) fragment.

**Preparation of F(ab')**_2_ **. **Sixty mg human IgG at pH: 3.20 were used for digestion with enzyme. Gel filtration separations of digested materials yielded 36 mg F(ab')_2_ fragment (Fig. 3). 

**Fig. 4:**
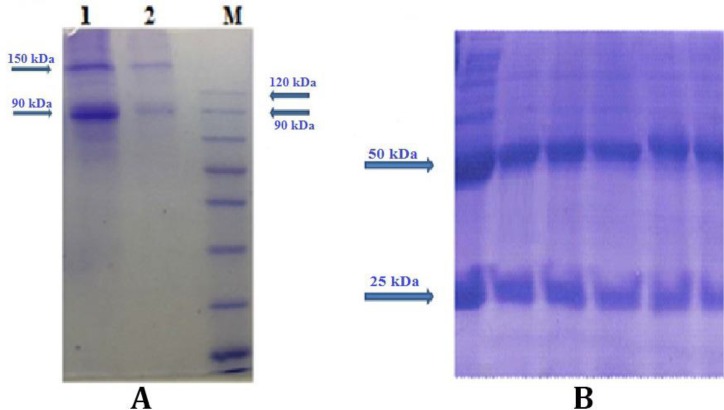
SDS- PAGE results for purity determination of purified F(ab')2. Lines 1 and 2: purified F(ab')_2_; M: Marker (A). SDS- PAGE analysis of rabbit polyclonal antibody (B).

The results of reduced SDS-PAGE for determining the purity of F(ab')_2_ fragment showed one band in 90 kDa corresponds to F(ab')_2 _fragment and a band in 150 kDa MW position corresponds to undigested whole IgG Abs (Fig. 4).

**Preparation of PA. **The serum of the immunized rabbit at 1/128000 dilution showed highest absorbance with purified F(ab')_2 _in designed ELISA method (Table 1). Protein content of the rabbit serum was 50 mg (3 mL). After precipitation and dialysis stages, 32 mg proteins were loaded on ion exchange chromatography column. Purification by ion exchange chromatography method resulted about 12 mg rabbit polyclonal anti-human IgG F(ab')_2 _Ab.

The results of reduced SDS-PAGE for determining the purity of polyclonal rabbit anti-human IgG F(ab')_2_ showed distinct band in 50 kDa and the bands between 25 to 30 kDa MW positions (Fig. 4).

**Table 1:** Evaluation of antibody production in the serum of immunized rabbit by ELISA.

**Dilution**	**Negative Control** *	**1/2000**	**1/4000**	**1/8000**	**1/16000**	**1/32000**	**1/64000**	**1/128000**	**1/256000**
**Optical Density**	0.12	> 3	2.73	2.45	2.15	1.89	1.45	1.13	0.92

* Negative control with 1/8000 dilution**.**

In addition, the purified Ab was conjugated with FITC. Next, the activities of FITC conjugated Ab and commercial ones were compared. According to flow cytometry results, generated PA had an acceptable reactivity compared to commercial Ab. Moreover, the produced Ab is efficiently effective and functional for use in applications such as flow cytometry.

## Discussion

In the present study, Ab sources were human and rabbit sera. Mammalian sera are notable sources of Abs. As a source of IgG, human sera were used. The IgG is a major component of mammalian serum immuno-globulins. Rabbit serum normally contains appreciable quantities of IgG.^6^

Based on published literature, IgG should be 15.00% or higher in the serum for efficient purification via ion exchange chromatography method. Due to high binding capacity and cost-effectiveness, ion exchange chromatography was used for purification of human and rabbit IgG. The purification of Abs by this method are related to several factors such as buffer type, charge, pH and ionic strength.^21^^-^^24^ In present study by changing the mobile phase, the polyclonal rabbit Ab was eluted. This method was well established in our laboratory for the purification of the IgG Ab. 

In similar previous study, Ab F(ab')_2_ fragments were purified in high yield from serum which could be used in designing ELISA kits.^7^

According to the SDS-PAGE results, purified F(ab')_2_ fragments obtained with purity higher than 95.00%. Some other studies lack appropriate control methods evaluating the activity of purified products.^25^ In present study, the activity of purified F(ab')_2_ fragments was evaluated. For investigation of activity of purified F(ab')_2_ fragments and production of PA against them, purified F(ab')_2_ fragments were injected to rabbit and rabbit immunity responses were studied. 

Virus inactivation of injected antigen is an important step in immunization processes. This study provides significant virus clearance because pH of elution stage in ion exchange chromatography stage and used optimum pH for pepsin digestion are close to the desirable pH for virus inactivation.^26^

For production of PA, it is important to consider antigen quality and quantity. The specificity and high titer of obtained rabbit anti F(ab')_2_ fragment depend on purity of the injected antigen.^27^^-^^29^

Therefore, in this study for induction of effective Ab responses, highly purified human F(ab')_2_ fragment was used to immunize rabbit and animal was given injections of antigen or antigen/adjuvant mixtures in various points of body. Upon such procedure, high immunologic response in rabbit was obtained, whose serum resulted in absorbance above 1 at 1/128000 dilution.

Polyclonal IgG Abs are involved predominantly in secondary immune response,^30^^,^^31^ therefore, the antigen injections in present study were repeated.

In this study, rabbit was not encountered with infectious agents that may suppress, modulate or stimulate its immune system. Veterinarian considered the health of rabbits by daily monitoring of their body temperatures.

According to the SDS-PAGE results, rabbit IgG obtained with purity higher than 95%. Product purity in present study was higher in comparison with previous studies.^21^

According to other studies in our laboratory, it is likely that diffused bands of light chain in SDS-PAGE results (Fig. 4) could be related to different levels of deglycosilation of protein during manipulation process.^12^

Purification by this method yielded about 12 mg of rabbit polyclonal IgG which was more than one third of the primary protein content. Product amount and output of this method were higher compared to previous reports.^6^^,^^19^^,^^22^

The ELISA method was done and the results showed that harvested PA recognizes human IgG F(ab')_2_ fragment. Furthermore, anti-IgG F(ab')_2 _PA was interacted with human IgG F(ab')_2_ fragment with a very high specificity and affinity. Polyclonal anti- human IgG F(ab')_2_ is very important and significant and it is a key reagent for its recognition. The PAs with high affinity are useful tools in biomedical and biochemical researches. For example, they can be used as ligands for the preparation of immunoaffinity columns for IgG F(ab')_2 _purification.^32^ Also, it is applicable in immunoassay tests for detection and quantitation of human IgG subclass and human IgG F(ab')_2_ fragment levels in immunoassay tests such as flow cytometry, ELISA, western blot, immunofluorescence, immunohistochemistry, immunoelectrophoresis and immunodiffusion tests.^5^^,^^33^^,^^34^

In conclusion, purified IgG F(ab')_2 _is applicable for conjugation with enzymes and radiolabels. This product is applicable in immunoelectrophoresis, immuno-diffusion, immunohistochemistry and flow cytometric tests and as immunoadsorbant for attachment to solid supports. This product is suitable for use as a primary reagent in enzyme immunoassays, western blot and cell or tissue immunostaining. The F(ab')_2_ fragments are recommended for staining of cells or tissues which contain Fc receptors.^13^^,^^35^^,^^36^
